# Spatial Investigation of Facet Joint Cartilage Properties Reveals Specific Structure–Function Relationships

**DOI:** 10.1002/jsp2.70209

**Published:** 2026-07-31

**Authors:** Luisa de Roy, Ann‐Kathrin Greiner‐Perth, Graciosa Quelhas Teixeira, Benjamin Mayer, Hans‐Joachim Wilke, Andreas Martin Seitz, Christian Liebsch

**Affiliations:** ^1^ Institute of Orthopaedic Research and Biomechanics, Centre for Trauma Research, Ulm University Medical Centre Ulm Germany; ^2^ Institute for Epidemiology and Medical Biometry, Ulm University Ulm Germany

**Keywords:** biomechanics, cartilage, facet joint, function, in vitro, structure

## Abstract

**Background:**

Facet joints are highly susceptible to osteoarthritis, which can manifest in distinct patterns of cartilage degeneration across the facet joint surfaces. Understanding how cartilage structure translates into functional properties of the tissue may help to identify regions that are, from a biomechanical perspective, at higher risk for degenerative changes. Therefore, this in vitro study aimed to determine structure–function relationships based on spatially assessed surface properties of intact facet joint cartilage.

**Methods:**

Biomechanical properties were assessed by spatial stress‐relaxation tests, which were performed at 12 measurement points on the cartilage surfaces of six inferior and six superior ovine facets, respectively. Cartilage thickness was determined at corresponding locations by the needle penetration method. Spatial histological analyses were performed to assess collagen and glycosaminoglycan contents, quantified from Picrosirius‐red‐ and Safranin‐O‐stained sections, respectively. Differences between inferior and superior facets were analyzed using linear‐mixed models; structure–function relationships were analyzed using Spearman's correlation.

**Results:**

Cartilage thickness and glycosaminoglycan coverage were significantly higher at superior facets (*p* < 0.05). Biomechanical properties did not significantly differ between inferior and superior facets (*p* ≥ 0.05). The relaxation behavior indicated a significant (*p* < 0.05) positive correlation with cartilage thickness at inferior facets (*ρ* = 0.606) and with glycosaminoglycan coverage at superior facets (*ρ* = 0.597).

**Conclusion:**

The biomechanical behavior of intact ovine facet cartilage was associated with its structure and the assessed properties indicated spatial variation across the facet joint surface. Overall, this study highlights the importance of topographic characterization of human facet cartilage to better understand facet osteoarthritis.

## Graphical abstract

The inferior facet of the L2 vertebra and the superior facet of the L3 vertebra were harvested from ovine lumbar spines. The spatial biomechanical properties of the facet cartilage surfaces were determined by indentation mapping. Cartilage structure was spatially analyzed on a macroscopic and microscopic level. Structure‐function relationships were subsequently evaluated by correlating the assessed biomechanical and structural parameters. All outcome measures varied across the facet joint surface, and the biomechanical properties were significantly associated with cartilage structure.

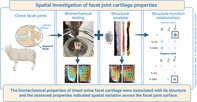

## 1 Introduction

The facet joints are highly susceptible to early osteoarthritis, which is considered a potential cause of low back pain due to their rich innervation [1, 2]. Facet joint osteoarthritis involves progressive structural and functional alterations of the cartilage that covers the facet joint's inferior and superior articular process [1, 2, 3]. Despite extensive research, the complex pathophysiology of cartilage degeneration remains incompletely understood [3, 4, 5]. Consequently, long‐term joint‐preserving or curative treatment of facet joint osteoarthritis remains a challenge [4, 5]. One key pathogenic factor implicated in osteoarthritis development is chronic mechanical overloading of the facet cartilage [4]. Alterations in the biomechanical load‐bearing properties may activate catabolic processes in the mechanosensitive tissue, which in the long term contribute to the progressive cartilage degradation identified in osteoarthritis [4, 6, 7]. So far, it remains unclear whether the inferior or superior facet process is generally more prone to degeneration [5, 8]. Moreover, the literature suggests that topographical aspects may play a role in disease development, as the extent of cartilage degeneration varies across the facet joint surfaces. Tischer et al. demonstrated that structural damage occurs particularly in regions exposed to the highest mechanical loading during maximal flexion and extension [2]. This raised the question of whether specific regions on cartilage surfaces are more susceptible to degeneration due to local differences in biomechanical load‐bearing capacity. Such differences may be related to topographic variations in tissue structure, as cartilage functionality strongly depends on its biphasic composition, consisting of a solid collagen–proteoglycan matrix and an interstitial fluid phase [9, 10]. However, topographical heterogeneity in facet joint cartilage structure and function remains largely unaddressed so far, leaving a critical knowledge gap. Therefore, the objective of this study was to compare structural and biomechanical properties of intact facet joint cartilage surfaces and to determine structure–function relationships based on spatially assessed cartilage properties. For the first time, mapping the biomechanical and structural properties across the cartilage surfaces provided insights into their topographical heterogeneity. Overall, characterizing the properties of healthy facet joint cartilage is fundamental, as it establishes the baseline required to interpret pathological changes in facet osteoarthritis and to advance the development of targeted therapeutic strategies.

## 2 Material and Methods

### 2.1 Study Design

**FIGURE 1:**
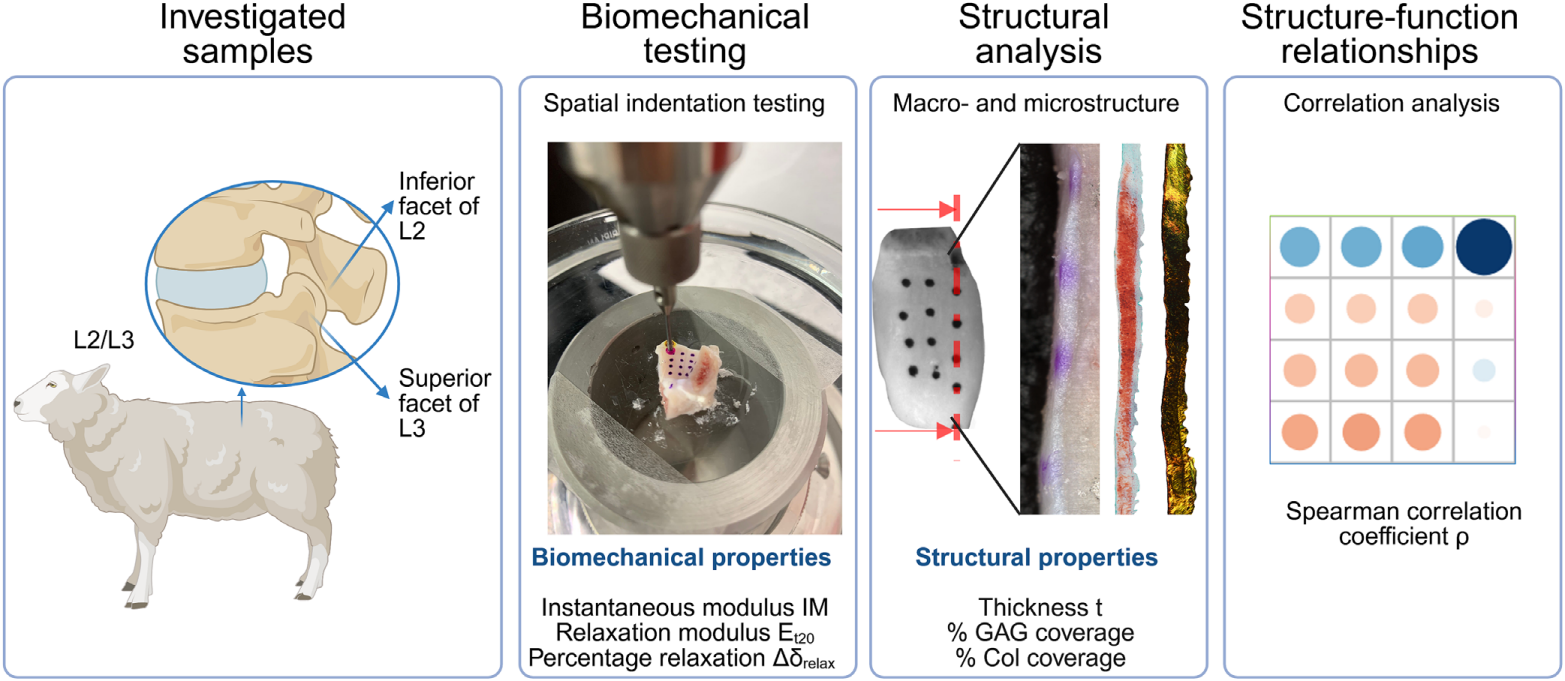
Schematic overview of the study design. The inferior facet of the L2 vertebra and the superior facet of the L3 vertebra were harvested from six ovine lumbar spines. The biomechanical properties of the facet cartilage surfaces were investigated by indentation mapping. Cartilage structure was analyzed on a macroscopic and microscopic level. Correlation analyses were performed to identify relationships between the structural and biomechanical properties of the cartilage surfaces. Created in BioRender [11].

For this in vitro study, six macroscopically intact left facet joints were extracted from six fresh frozen ovine lumbar spines (female merino sheep, ~2.5 years old) (Figure 1). Spatial normal indentation tests were performed at 12 measurement points on the cartilage surface of the inferior facet of the L2 vertebra and the superior facet of the L3 vertebra to determine the biomechanical properties (Figure 1). The thickness of the cartilage at each measurement point was determined as a macroscopic structural parameter. As microscopic structural parameters, Safranin‐O and Picrosirius‐red stained tissue sections were analyzed to quantify the relative glycosaminoglycan and collagen coverage, respectively. Differences between inferior and superior facets were analyzed using linear‐mixed model (LMM) analyses, while relationships between structural and biomechanical properties were assessed using Spearman's correlation method.

### 2.2 Specimen Preparation

To obtain the left facet joints, the vertebral bodies of L2 and L3 were separated by dissecting the intervertebral disc, followed by careful opening of the facet joint capsule using a scalpel. After disarticulating the facet joints, a diamond band saw (Exakt 300, Exakt Technologies Inc., Oklahoma, USA) was used to isolate the inferior facet of the L2 vertebra and the superior facet of the L3 vertebra, respectively. Throughout specimen preparation, care was taken to avoid damage to the cartilage surfaces and to maintain tissue hydration using phosphate buffered saline solution.

### 2.3 Biomechanical Testing

The biomechanical properties of the facet joint cartilage were determined by spatial indentation testing, which was performed at 12 measurement points at inferior and superior, respectively. The position of the measurement points was standardized and marked with a surgical marking pen (Devon, Cardinal Health, Dublin, Ireland). At each point, a non‐destructive normal indentation stress‐relaxation test was carried out using a multiaxial mechanical testing machine (MACH‐1 v500css, Biomomentum Inc., Laval, QC, Canada). The machine was equipped with a multiaxial 70 N load cell (ATI Industrial Automation, Apex, USA) and a spherical indenter with a diameter of 2 mm. The bony part of the facets was glued on a sample holder for fixation in the testing machine and kept hydrated with phosphate‐buffered saline during testing. The following test parameters were applied: contact criteria 0.1 N, indentation amplitude 0.08 mm, indentation velocity 0.08 mm/s, relaxation time 20 s. Needle penetration testing was used to determine the cartilage thickness at each measuring point (26‐G 3/8‐in. intradermal bevel needle, BD, Franklin Lakes, NJ, USA). Finally, the instantaneous modulus in MPa as a measure of the initial elastic response (Equation 1) and the relaxation modulus *E*_*t*20_ in MPa as a measure of the initial viscous (Equation 2) response were calculated [12, 13]:

(1) $$\mathrm{IM}=\frac{F}{H}\times \frac{1-v^{2}}{2\mathrm{a\kappa}\;\left(\frac{a}{t,v}\right)}$$

with the resulting force *F* in N, the indentation amplitude *H* in mm, the Poisson's ratio *ν* = 0.4, and the cartilage thickness *t* in mm. *a* is defined as the radius of the spherical indentation segment of the indenter at the indentation amplitude in mm. κ is a correction factor set by the software (Automated Indentation and Thickness Batch Analysis tool, Biomomentum Inc., Laval, QC, Canada) automatically depending on *a*/*t* and *ν*:

(2) $$E_{t20}=\frac{F_{t20}/A_{\text{sseg}}}{H/t}$$

with the force at the end of the relaxation time *F*_*t*20_ in N and *A*_sseg_ in mm^2^ being the resultant area of the spherical indentation segment.

In addition, the stress relaxation behavior ∆*δ*_relax_ in % was determined to obtain further insight into the viscoelasticity of the cartilage (Equation 3) [13]:

(3) $$∆\delta_{\text{relax}}=\frac{\delta_{t20}}{\delta_{\max}}\times 100\%$$

whereby *δ*_*t*20_ is the stress at the end of the relaxation time and *δ*_max_ is the maximum stress in MPa.

### 2.4 Structural Analysis

**FIGURE 2:**
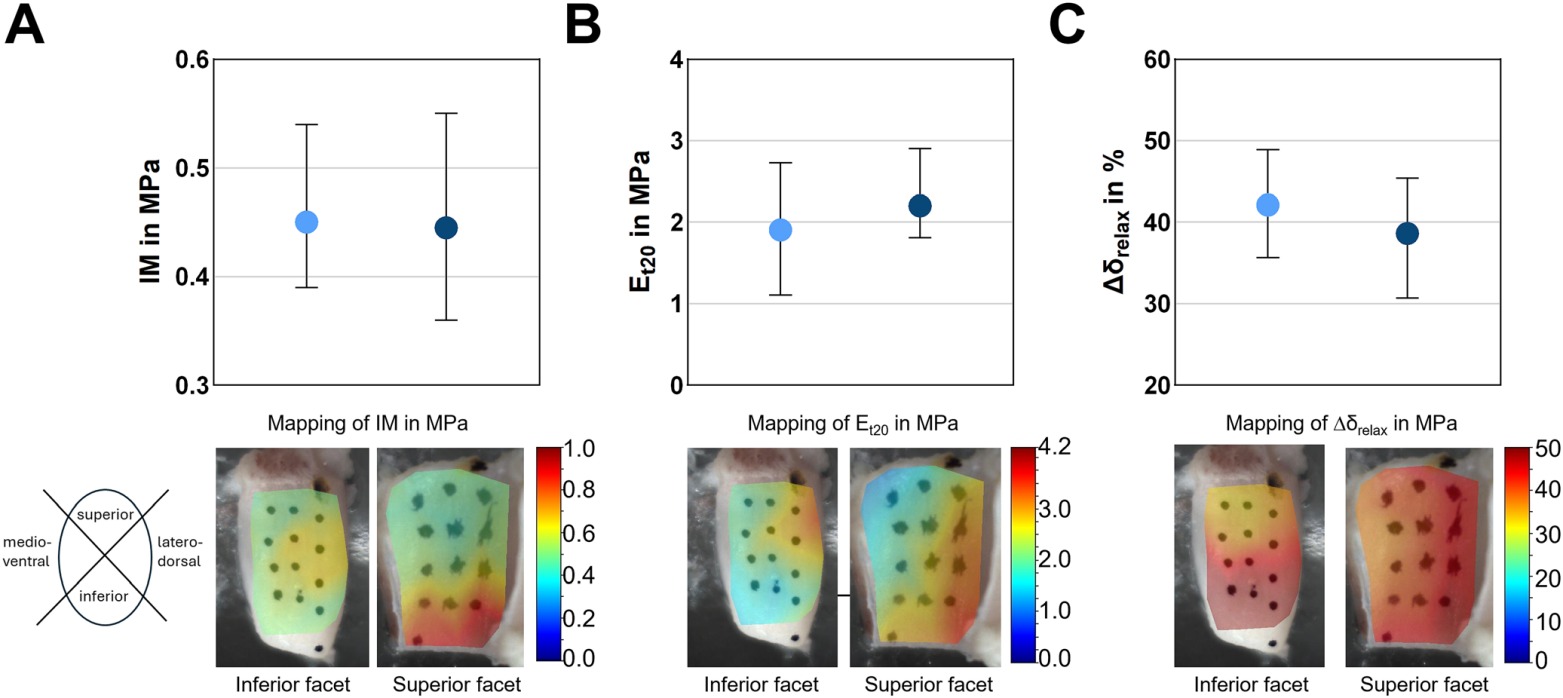
Results of biomechanical testing, shown as median with 95% confidence intervals. (A) Instantaneous modulus (IM in MPa) as a measure of the initial elastic response, (B) relaxation modulus at 20 s relaxation (*E*_*t*20_ in MPa) as a measure of the initial viscous response, and (C) Δ*δ*_relax_ in % as a measure of the viscoelastic behavior. Results of the inferior facet are shown in light blue, those of the superior facet in dark blue. For each parameter, the spatial distribution on the respective facet surface was visualized in mappings displaying the median values of the six specimens at each measurement point. Scales are displayed from 0 to the maximum value of each parameter. Differences evaluated by linear mixed model analyses with *n* = 72 data points.

**FIGURE 3:**
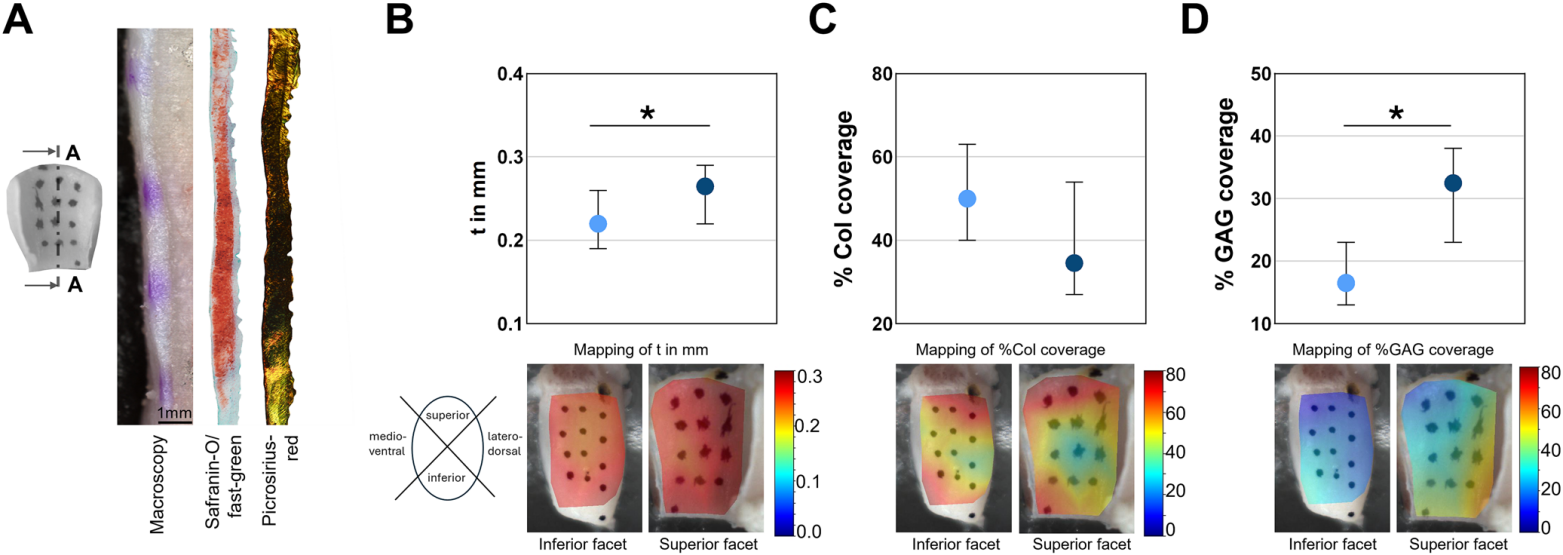
Results of structural properties. (A) Representative macroscopic image and microscopic stainings on the example of superior facet cartilage. (B) Cartilage thickness (*t* in mm), (C) relative area of collagen (% Col coverage), and (D) relative area of glycosaminoglycan (% GAG coverage). Results shown as median with 95% confidence intervals, for the inferior facet in light blue, for the superior facet in dark blue. For each parameter, the spatial distribution on the respective facet surface was visualized in mappings displaying the median values of the six specimens at each measurement point. Scales are displayed from 0 to the maximum value of each parameter. * indicates significant difference (*p* < 0.05), evaluated by linear mixed model analyses with *n* = 72 data points.

After biomechanical testing, each facet was cut along the investigated measurement points (superior to inferior direction) using a diamond band saw. Macroscopic images of the cut surfaces were acquired using a digital microscope (Keyence VHX, Keyence Deutschland GmbH, Frankfurt, Germany). For histological analyses, the samples were fixed in saline‐buffered formalin for 48 h, followed by decalcification for 30 days in ethylenediaminetetraacetic acid. Decalcified samples were embedded in paraffin and sections with a thickness of 6 μm were prepared. Safranin‐O/fast‐green staining was performed to evaluate glycosaminoglycan content [14]. Picrosirius‐red staining was used to analyze the collagen content [15]. Safranin‐O‐stained sections were imaged by light microscopy (Zeiss Axiophot, Zeiss, Oberkochen, Germany) under the same illumination conditions in a single session. Picrosirius‐red stained sections were imaged with polarized light filters, with all images captured with the same parameters. Image analysis using Image J with the Fiji package (NIH, Bethesda, MD) was performed to quantify the glycosaminoglycan and collagen areas (in %) from the stained sections. In order to determine the composition at each measurement point, the macroscopic and microscopic images of the facets were matched in Fiji first. Then, a region of interest (ROI; ~2 mm in width, corresponding to indenter diameter) was defined on the microscopic images based on the measurement points visible on the macroscopic images. The percentage of glycosaminoglycans in the ROI was derived from the Safranin‐O‐stained sections using the color deconvolution tool (% GAG coverage). The percentage of collagen in the ROI was determined as the sum of red (1–9 nm; 230–255 nm), orange (10–38 nm), yellow (39–51 nm) and green (52–128 nm) fibers in the picrosirius red stained sections following Widmayer et al. [16] (% Col coverage). For spatial visualization of the results, the median value at each measurement point was determined and mapped on a representative image of an inferior and superior facet using Kriging interpolation. For qualitative comparisons, each surface was subdivided into anatomical regions as described by Tischer et al. (Figures 2 and 3) [2].

### 2.5 Statistical Analysis

Descriptive results were presented using median with 95% confidence interval (CI). Differences in assessed outcome parameters between inferior and superior facets were analyzed using LMM analyses. Specifically, LMMs included the main effects of type of facet (inferior vs. superior) and measurement point (1–12) as well as their interaction as fixed effects, and also a random intercept in order to account for multiple measures from the same sample (sheep). Estimation of statistical significance (inferior/superior comparison) was based on model‐based, Tukey‐adjusted linear contrast hypotheses. Correlations between the structural and biomechanical properties were evaluated using the Spearman rank correlation method (correlation coefficient ρ). Statistical analyses were conducted using GraphPad Prism 10 (GraphPad Software, La Jolla, CA, USA) and R (version 4.5.0; www.r‐project.org, packages *lmer* and *emmeans*).

## 3 Results

### 3.1 Functional Properties

No significant differences in the instantaneous modulus were observed when comparing inferior facets (median with 95% CI: 0.45 [0.39, 0.54]) with superior facets (0.45 [0.36, 0.55]) (*p* ≥ 0.05, Figure 2A). Visualization of the median instantaneous modulus at each measurement point demonstrated that the inferior region of the superior facet indicated the highest elastic response. E_t20_ values of inferior facets (1.92 [1.12, 2.73]) and superior facets (2.20 [1.81, 2.90]) did not significantly differ (*p* ≥ 0.05, Figure 2B). *E*_*t*20_‐mappings revealed that the latero‐dorsal regions on both facet surfaces exhibited the highest viscous response compared to medial‐ventral and superior regions. The results of ∆*δ*_relax_ did not show a significant difference between inferior facets (42 [36, 49]) and superior facets (37 [31, 45]) (*p* ≥ 0.05, Figure 2C). The spatial distribution of Δ*δ*_relax_ showed the lowest values in the superior region of the inferior facets.

### 3.2 Structural Properties

Cartilage was significantly thicker at superior facets (0.27 [0.24, 0.28]) compared to inferior facets (0.22 [0.21, 0.25]) (*p* = 0.023, Figure 3B). Mapping the spatial distribution of t showed a homogeneous thickness across both facet surfaces. No significant differences were found in the relative collagen (% Col) coverage when comparing inferior facets (50 [41, 56]) and superior facets (35 [34, 48]) (*p* ≥ 0.05, Figure 3C). % Col mapping indicated that the cartilage in the superior and inferior regions of the facet surfaces showed the highest collagen coverage. A significantly higher relative glycosaminoglycan (% GAG) coverage was found in the cartilage at superior facets (33 [28, 40]) compared to inferior facets (17 [16, 23]) (*p* = 0.004, Figure 3D). Spatial visualization of the % GAG coverage showed that the highest values were observed in the latero‐dorsal region of superior facets.

### 3.3 Correlation Analysis of Structural and Functional Properties

**FIGURE 4:**
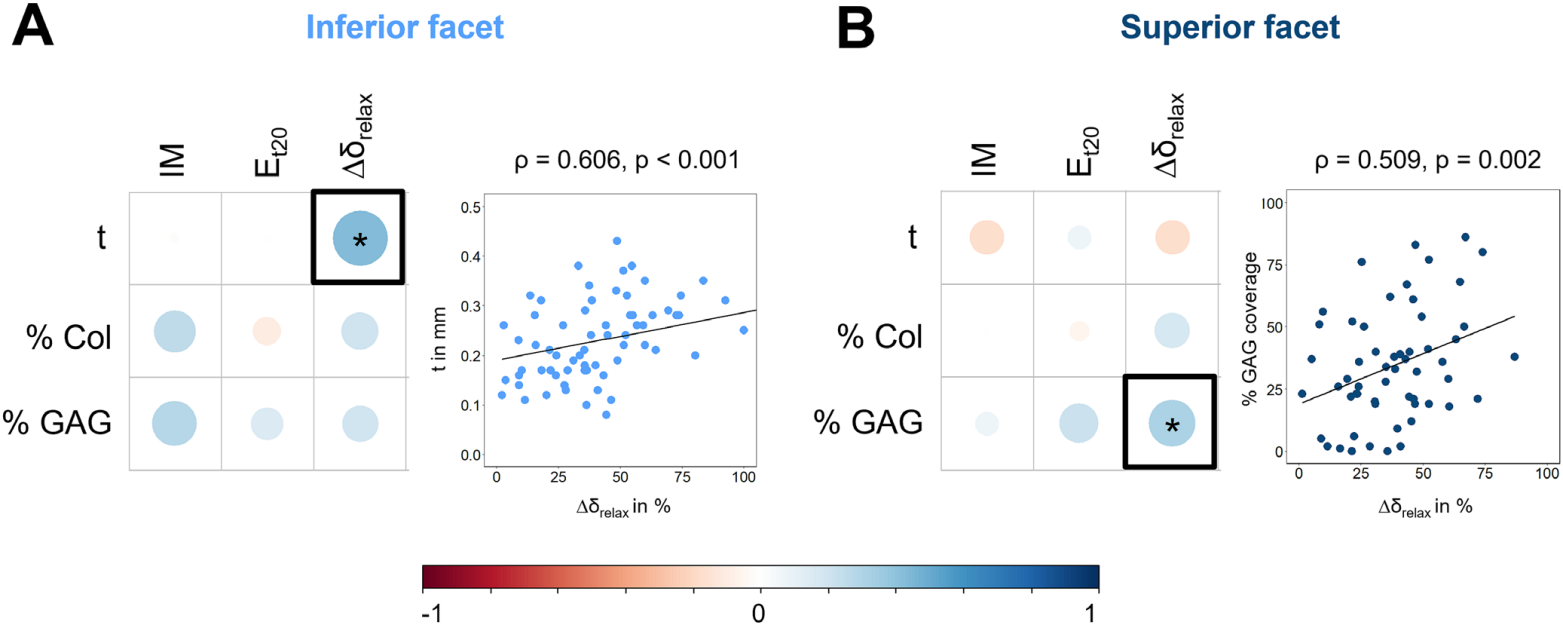
Correlations between structural and biomechanical properties of (A) inferior and (B) superior facet joint cartilage. Spearman correlation matrix showed positive correlations in blue, negative correlations in red. Color intensity and the size of the circle are proportional to the correlation coefficient. *n* = 72 pairwise comparisons were performed, * and black squares in the matrix indicate statistically significant correlation (*p* < 0.05). Scatter plots with linear regression of significant correlations are shown.

For inferior facets, a significant positive correlation (*ρ* = 0.606, *p* < 0.001) was found between t and Δ*δ*_relax_ (Figure 4A). At superior facets, higher % GAG coverage showed a strong relation to higher Δ*δ*_relax_ values, as indicated by a significant positive correlation between these two parameters (*ρ* = 0.597, *p* = 0.002, Figure 4B).

## 4 Discussion

This study analyzed structure–function relationships of intact facet joint cartilage surfaces and provided insights into spatial distribution of the assessed structural and biomechanical properties. The most important finding was that the biomechanical behavior of the cartilage of both the inferior and superior facets was significantly associated with their structural properties. Since cartilage is a highly mechanoadaptive tissue in which mechanical stimuli regulate extracellular matrix synthesis and maintain tissue homeostasis [17, 18], this may represent a response to facet joint loading. While axial compressive loads have been shown to be transmitted through the facet joints [19, 20, 21], the exact mechanisms of load transfer have not been fully elucidated [22]. Foil sensors have been used to assess pressure distribution on the facet surfaces ex vivo [21]. However, these measurements may be influenced by the invasiveness of sensor insertion into the articulating space, thereby potentially altering native joint contact mechanics. The remaining knowledge gap about load distribution in the facets not only remains a challenge in understanding facet joint degeneration but also in modeling the facet joints in finite element simulations of the spine [23]. This represents a significant limitation, given their important role in guiding spinal motion. For the first time, spatial visualization of the structural and biomechanical properties provided stratified insights throughout the cartilage surfaces. The mappings indicated local variations, which may reflect a dependence of cartilage function on its structural composition [9]. Interestingly, highest collagen content was found in the inferior and superior regions on both the inferior and superior facet. Based on the mechanoadaptive nature of cartilage, it can be hypothesized that different loading patterns during spine motion may lead to heterogeneity in facet cartilage structure and consequently region‐specific cartilage biomechanics. During flexion movement, the inferior facets of the superior vertebrae cause maximum pressure on the superior region of the superior facets of the inferior vertebrae, whereas contact pressure between the inferior region of the inferior facet and the superior region of the superior facet can increase during extension movement [2, 19, 24, 25]. Increased collagen content in these areas may contribute to adequate biomechanical properties necessary to withstand high loading during flexion/extension. This aligns with high instantaneous modulus values found in these regions. This biomechanical parameter reflects the immediate elastic response of the collagen matrix before fluid pressurization contributes to the time‐dependent load‐bearing capacity [13, 26]. It can be speculated that these regions are at higher risk to be impaired by cartilage degeneration, which is characterized by disruption of the collagen network [13, 27]. If degenerative changes occur in these areas, the cartilage may be unable to withstand high pressures, which then potentially trigger catabolic, matrix‐degrading pathways leading to cartilage degradation over time [6, 28]. In degenerated human facets, it has been shown that cartilage damage is particularly evident in the inferior and superior areas across the facet surface [2]. This might be the result of local excessive overloading, as load distribution is considered a major factor in the development and progression of facet joint osteoarthritis [2, 3]. An early sign of cartilage degeneration is GAG depletion [29]. This is critical as glycosaminoglycans play a key role in regulating cartilage permeability by promoting water retention within the solid matrix, an important feature for the time‐dependent load support of the tissue [9, 29]. In degenerated cartilage, reduced glycosaminoglycan content is associated with faster fluid flow, which compromises the stress relaxation behavior and thus the time‐dependent load support [13, 27]. In this context, the presented correlation data might suggest that the cartilage at the superior facet is overall more susceptible to early biomechanical changes, as the glycosaminoglycan content was associated with the stress‐relaxation behavior at this level. At inferior facets, in contrast, the biomechanical impairment might manifest more prominently in later stages of facet joint osteoarthritis, when cartilage thinning is already macroscopically evident [5, 30]. Knowledge of structure–function relationships might also be relevant from a clinical perspective, because compositional parameters such as glycosaminoglycan content and thickness can be visualized using magnetic resonance imaging techniques [30, 31]. Correlating the structural and biomechanical properties of facet cartilage might enable in vivo assessment of facet joint functionality and its potential association with pain in the future [32]. Such approaches offer the possibility to provide valuable insights into how altered spinal motion and loading conditions, for example following intervertebral disc arthroplasty or spinal fusion, contribute to adjacent facet joint degeneration [33]. Moreover, spatial assessment of facet cartilage structure and function may provide useful outcome measures for future studies evaluating targeted facet joint interventions, including intra‐articular injections of orthobiologics or other disease‐modifying treatment strategies [34].

Overall, the presented findings extend the current literature by providing data on differences between opposing ovine facet joints, demonstrating significant differences in structural properties of the cartilage between inferior and superior facets. These findings contrast with findings from O'Leary et al., who reported no difference in thickness and glycosaminoglycan and collagen content between opposing facet surfaces in rabbits and mini‐pigs [8]. However, in their study, cartilage composition was analyzed biochemically at one central location, which might explain the differences in findings compared to the present study. Moreover, the biomechanical properties of the inferior and superior facet processes did not significantly differ in the present study, which is in agreement with studies on monkey, rabbit, and mini pig facets [8], as well as on human facets [1].

This study has limitations that need to be addressed. First, facet joint size, shape, and consequently the loading in quadrupedal animals is different from those in bipedal humans [5, 35, 36]. Therefore, the herein presented findings of ovine facets cannot directly be translated to humans, requiring investigations of human facet joints in both healthy and degenerated conditions to improve clinical translatability. Nevertheless, the ovine model has been considered suitable for spine research due to biomechanical similarities between sheep and humans [36, 37] and provides the advantage of less variability regarding morphology and degenerative alterations compared to human specimens. Second, the correlation analysis was performed without regional stratification to ensure sufficient statistical power. Stratification of different areas across the facet surface in upcoming correlation studies may improve the understanding of regional differences in structure–function relationships. Third, the analysis was restricted to facet joints from L2 to L3 motion segments. Care should be taken when extrapolating the findings to the entire spine because spinal loading likely varies by spinal level [38]. While investigating facets from animals revealed no level‐dependent differences in the biomechanical properties in the present as well as a previous study [8], Gupta et al. demonstrated an increased susceptibility to degeneration at the lower lumbar levels in humans (L3–S1) [1]. Consequently, future studies on human samples should account for level dependent analyses.

In conclusion, this in vitro study revealed that the biomechanical properties of intact ovine facet joint cartilage were associated with their structural properties and that these properties indicated spatial variation across the facet joint surfaces. Moreover, significant differences in structural properties between the inferior and superior facet were determined, while no significant differences in biomechanical properties were found. Overall, these findings highlight the importance of topographic characterization of facet joint cartilage in healthy and degenerative conditions. Applying the presented methodological framework to human facet joints in future studies has the potential to provide important insights into the pathophysiology of facet joint osteoarthritis.

## Author Contributions

**Luisa de Roy:** conceptualization, methodology, investigation, formal analysis, writing – original draft. **Ann‐Kathrin Greiner‐Perth:** resources, methodology, writing – review and editing. Luisa de Roy: conceptualization, methodology, investigation, formal analysis, writing – original draft. **Graciosa Quelhas Teixeira:** resources, methodology, writing – review and editing. **Benjamin Mayer:** writing – review and editing, formal analysis, data curation. **Hans‐Joachim Wilke:** writing – review and editing, conceptualization, supervision, funding acquisition. **Andreas Martin Seitz:** conceptualization, supervision, funding acquisition, methodology, writing – review and editing. **Christian Liebsch:** conceptualization, supervision, project administration, writing – review and editing.

## Conflicts of Interest

The authors declare no conflicts of interest.

## Data Availability

The data that support the findings of this study are available from the corresponding author upon reasonable request.

## References

[1] S. Gupta , R. Xiao , M. Fainor , R. L. Mauck , H. E. Smith , and S. E. Gullbrand , “Level Dependent Alterations in Human Facet Cartilage Mechanics and Bone Morphometry With Spine Degeneration,” Journal of Orthopaedic Research 41, no. 3 (2023): 674–683, 10.1002/jor.25407. 35770853 PMC9800647

[2] T. Tischer , T. Aktas , S. Milz , and R. V. Putz , “Detailed Pathological Changes of Human Lumbar Facet Joints L1‐L5 in Elderly Individuals,” European Spine Journal 15, no. 3 (2006): 308–315, 10.1007/s00586-005-0958-7. 16021481 PMC3489294

[3] A. C. Gellhorn , J. N. Katz , and P. Suri , “Osteoarthritis of the Spine: The Facet Joints,” Nature Reviews Rheumatology 9, no. 4 (2013): 216–224, 10.1038/nrrheum.2012.199. 23147891 PMC4012322

[4] W. Van Oosterwyck , P. Vander Cruyssen , F. Castille , E. Van de Kelft , and V. Decaigny , “Lumbar Facet Joint Disease: What, Why, and When?,” Life 14, no. 11 (2024): 1480, 10.3390/life14111480. 39598277 PMC11595282

[5] S. A. O'Leary , N. K. Paschos , J. M. Link , E. O. Klineberg , J. C. Hu , and K. A. Athanasiou , “Facet Joints of the Spine: Structure‐Function Relationships, Problems and Treatments, and the Potential for Regeneration,” Annual Review of Biomedical Engineering 20 (2018): 145–170, 10.1146/annurev-bioeng-062117-120924. 29494214

[6] W. Lin and J. Klein , “Recent Progress in Cartilage Lubrication,” Advanced Materials 33, no. 18 (2021): e2005513, 10.1002/adma.202005513. 33759245

[7] G. Musumeci , “The Effect of Mechanical Loading on Articular Cartilage,” Journal of Functional Morphology and Kinesiology 1 (2016): 154–161, 10.3390/jfmk1020154.

[8] S. A. O'Leary , J. M. Link , E. O. Klineberg , J. C. Hu , and K. A. Athanasiou , “Characterization of Facet Joint Cartilage Properties in the Human and Interspecies Comparisons,” Acta Biomaterialia 54 (2017): 367–376, 10.1016/j.actbio.2017.03.017. 28300721

[9] A. J. S. Fox , A. Bedi , and S. A. Rodeo , “The Basic Science of Articular Cartilage: Structure, Composition, and Function,” Sports Health 1, no. 6 (2009): 461–468, 10.1177/1941738109350438. 23015907 PMC3445147

[10] C. G. Armstrong and V. C. Mow , “Variations in the Intrinsic Mechanical Properties of Human Articular Cartilage With Age, Degeneration, and Water Content,” Journal of Bone and Joint Surgery. American Volume 64, no. 1 (1982): 88–94. 7054208

[11] W. Ohmayer , “Study Design,” 2026, https://BioRender.com/ood999s.

[12] W. C. Hayes , L. M. Keer , G. Herrmann , and L. F. Mockros , “A Mathematical Analysis for Indentation Tests of Articular Cartilage,” Journal of Biomechanics 5, no. 5 (1972): 541–551, 10.1016/0021-9290(72)90010-3. 4667277

[13] A. M. Seitz , F. Osthaus , J. Schwer , et al., “Osteoarthritis‐Related Degeneration Alters the Biomechanical Properties of Human Menisci Before the Articular Cartilage,” Frontiers in Bioengineering and Biotechnology 9 (2021): 659989, 10.3389/fbioe.2021.659989. 34026741 PMC8134692

[14] K. M. Fischenich , J. Lewis , K. A. Kindsfater , T. S. Bailey , and T. L. Haut Donahue , “Effects of Degeneration on the Compressive and Tensile Properties of Human Meniscus,” Journal of Biomechanics 48, no. 8 (2015): 1407–1411, 10.1016/j.jbiomech.2015.02.042. 25770751

[15] C. M. López De Padilla , M. J. Coenen , A. Tovar , R. E. De la Vega , C. H. Evans , and S. A. Müller , “Picrosirius Red Staining: Revisiting Its Application to the Qualitative and Quantitative Assessment of Collagen Type I and Type III in Tendon,” Journal of Histochemistry and Cytochemistry 69, no. 10 (2021): 633–643, 10.1369/00221554211046777. 34549650 PMC8504258

[16] F. Widmayer , C. Neidlinger‐Wilke , F. Witz , et al., “Oestrogen and Vibration Improve Intervertebral Disc Cell Viability and Decrease Catabolism in Bovine Organ Cultures,” International Journal of Molecular Sciences 24, no. 7 (2023): 6143, 10.3390/ijms24076143. 37047116 PMC10094023

[17] G. Wu , S. Chen , Q. Li , et al., “Mechanical Cues Enhance Chondrocyte Function: Insights From Mechanoreception, Regulation, and Biological Responses,” Mechanobiology in Medicine 3, no. 4 (2025): 100164, 10.1016/j.mbm.2025.100164. 41403648 PMC12704073

[18] T. L. Vincent and A. K. T. Wann , “Mechanoadaptation: Articular Cartilage Through Thick and Thin,” Journal of Physiology 597, no. 5 (2019): 1271–1281, 10.1113/jp275451. 29917242 PMC6395418

[19] R. B. Dunlop , M. A. Adams , and W. C. Hutton , “Disc Space Narrowing and the Lumbar Facet Joints,” Journal of Bone and Joint Surgery. British Volume 66, no. 5 (1984): 706–710, 10.1302/0301-620x.66b5.6501365. 6501365

[20] K. H. Yang and A. I. King , “Mechanism of Facet Load Transmission as a Hypothesis for Low‐Back Pain,” Spine 9, no. 6 (1984): 557–565, 10.1097/00007632-198409000-00005. 6238423

[21] J. P. Warren , E. Bomphrey , and M. Mengoni , “An Experimental Methodology to Measure the Effects of Intervertebral Interventions on the Facet Biomechanics In Situ,” Journal of Biomechanics 183 (2025): 112617, 10.1016/j.jbiomech.2025.112617. 40088554

[22] N. Inoue , A. A. E. Orías , and K. Segami , “Biomechanics of the Lumbar Facet Joint,” Spine Surgery and Related Research 4, no. 1 (2020): 1–7, 10.22603/ssrr.2019-0017. 32039290 PMC7002062

[23] M. Mengoni , “Biomechanical Modelling of the Facet Joints: A Review of Methods and Validation Processes in Finite Element Analysis,” Biomechanics and Modeling in Mechanobiology 20, no. 2 (2021): 389–401, 10.1007/s10237-020-01403-7. 33221991 PMC7979651

[24] L. A. Hadley , “Anatomico‐Roentgenographic Studies of the Posterior Spinal Articulations,” American Journal of Roentgenology, Radium Therapy, and Nuclear Medicine 86 (1961): 270–276. 13710344

[25] H. Schmidt , M. Bashkuev , M. Dreischarf , et al., “Computational Biomechanics of a Lumbar Motion Segment in Pure and Combined Shear Loads,” Journal of Biomechanics 46, no. 14 (2013): 2513–2521, 10.1016/j.jbiomech.2013.06.038. 23953504

[26] S. Sim , A. Chevrier , M. Garon , et al., “Non‐Destructive Electromechanical Assessment (Arthro‐BST) of Human Articular Cartilage Correlates With Histological Scores and Biomechanical Properties,” Osteoarthritis and Cartilage 22, no. 11 (2014): 1926–1935, 10.1016/j.joca.2014.08.008. 25168362

[27] L. de Roy , G. Q. Teixeira , J. Schwer , et al., “Structure‐Function of Cartilage in Osteoarthritis: An Ex‐Vivo Correlation Analysis Between Its Structural, Viscoelastic and Frictional Properties,” Acta Biomaterialia 190 (2024): 293–302, 10.1016/j.actbio.2024.10.027. 39427764

[28] T. Fang , X. Zhou , M. Jin , J. Nie , and X. Li , “Molecular Mechanisms of Mechanical Load‐Induced Osteoarthritis,” International Orthopaedics 45, no. 5 (2021): 1125–1136, 10.1007/s00264-021-04938-1. 33459826

[29] P. N. Bansal , N. S. Joshi , V. Entezari , M. W. Grinstaff , and B. D. Snyder , “Contrast Enhanced Computed Tomography Can Predict the Glycosaminoglycan Content and Biomechanical Properties of Articular Cartilage,” Osteoarthritis and Cartilage 18, no. 2 (2010): 184–191, 10.1016/j.joca.2009.09.003. 19815108

[30] A. Fujiwara , K. Tamai , M. Yamato , et al., “The Relationship Between Facet Joint Osteoarthritis and Disc Degeneration of the Lumbar Spine: An MRI Study,” European Spine Journal 8, no. 5 (1999): 396–401, 10.1007/s005860050193. 10552323 PMC3611192

[31] M. V. W. Zibetti , R. G. Menon , H. L. de Moura , X. Zhang , R. Kijowski , and R. R. Regatte , “Updates on Compositional MRI Mapping of the Cartilage: Emerging Techniques and Applications,” Journal of Magnetic Resonance Imaging 58, no. 1 (2023): 44–60, 10.1002/jmri.28689. 37010113 PMC10323700

[32] S. Brinkhof , R. Nizak , S. Sim , et al., “In Vivo Biochemical Assessment of Cartilage With gagCEST MRI: Correlation With Cartilage Properties,” NMR in Biomedicine 34, no. 3 (2021): e4463, 10.1002/nbm.4463. 33352622 PMC7900973

[33] I. Merem , R. Vasquez , J. Wise , et al., “A Review of Motion‐Preserving Cervical Spinal Implants and Fusion Constructs,” Bioengineering 13, no. 2 (2026): 228, 10.3390/bioengineering13020228. 41749767 PMC12938692

[34] R. Vekaria , R. Bhatt , D. R. Ellard , N. Henschke , M. Underwood , and H. Sandhu , “Intra‐Articular Facet Joint Injections for Low Back Pain: A Systematic Review,” European Spine Journal 25, no. 4 (2016): 1266–1281, 10.1007/s00586-016-4455-y. 26906169

[35] A. Bonilla , H. Iii , B. Gadomski , V. Patel , and T. Jeremiah , “Ovine Models of Intervertebral Disc Degeneration,” Annals of Translational Medicine 13 (2025): 79, 10.21037/atm-25-136. 41502426 PMC12771051

[36] H.‐J. Wilke , A. Kettler , and L. E. Claes , “Are Sheep Spines a Valid Biomechanical Model for Human Spines?,” Spine 22, no. 20 (1997): 2365–2374. 9355217 10.1097/00007632-199710150-00009

[37] M. Bashkuev , S. Reitmaier , and H. Schmidt , “Is the Sheep a Suitable Model to Study the Mechanical Alterations of Disc Degeneration in Humans? A Probabilistic Finite Element Model Study,” Journal of Biomechanics 84 (2019): 172–182, 10.1016/j.jbiomech.2018.12.042. 30660378

[38] A. G. Bruno , K. Burkhart , B. Allaire , D. E. Anderson , and M. L. Bouxsein , “Spinal Loading Patterns From Biomechanical Modeling Explain the High Incidence of Vertebral Fractures in the Thoracolumbar Region,” Journal of Bone and Mineral Research 32, no. 6 (2017): 1282–1290, 10.1002/jbmr.3113. 28244135 PMC5466490

